# Clinical efficacy of socket shield technique compared to conventional immediate implant placement in the aesthetic zone: a meta-analysis

**DOI:** 10.1186/s40729-025-00657-z

**Published:** 2025-12-19

**Authors:** Wei Lu, Shanshan Du, Jingtong Su, Yang Wu, Xinyu Yao, Chao Zhang, Hedong Yu

**Affiliations:** 1 Department of Stomatology, Taihe Hospital, Hubei University of Medicine, Shiyan, 442000 Hubei China; 2 Center for Evidence-Based Medicine and Clinical Research, Taihe Hospital, Hubei University of Medicine, Shiyan, 442000 Hubei China

**Keywords:** Evidence-based dentistry, Immediate implant placement, Socket shield technique, Aesthetic zone

## Abstract

**Objective:**

To systematically evaluate and compare the clinical effects of the socket shield technique (SST) and conventional immediate implant placement (CIIP) in the esthetic zone through meta-analysis.

**Methods:**

A systematic search was conducted in PubMed, EMBASE, Cochrane Library, Web of Science, China National Knowledge Infrastructure, Chinese Science and Technology Periodical Database (VIP), and Wanfang Database for studies comparing the clinical and aesthetic effects of SST and CIIP, with the retrieval period spanning from database inception to October 9, 2024. After independent literature screening, data extraction, and bias risk assessment were independently performed by two investigations according to inclusion and exclusion criteria. All data analyses were performed by RevMan 5.4 software.

**Results:**

A total of 27 studies, including 22 randomized controlled trials and 5 non-randomized studies of interventions (NRSI), involving 1307 implants, were included in the meta-analysis. Meta-analysis demonstrated that SST significantly outperformed CIIP in reducing horizontal buccal bone loss (MD = −0.50, 95%CI [−0.60, −0.41], I^2^ = 97%) and vertical buccal bone loss (MD = −0.56, 95%CI [−0.64, −0.48], I = 78%), as well as improving the pink esthetic score (PES: MD = 1.25, 95%CI [0.93, 1.57], I = 90%) and implant stability quotient (ISQ: MD = 5.83, 95%CI [4.08, 7.57], I^2^ = 69%). No significant difference was observed in implant success rate (RR = 1.00, 95% CI [0.98, 1.02], I^2^ = 0%). Subgroup analyses (the height and thickness of buccal shield, bone grafting, and publication language) aligned with primary outcome (horizontal buccal bone loss), and sensitivity analysis confirmed stable results.

**Conclusion:**

Based on the available evidence, SST demonstrated favorable outcomes in reducing buccal bone loss, enhancing esthetic outcomes and implant stability while maintaining comparable implant success rates to CIIP. Nevertheless, the technique exhibited technical sensitivity and a lack of standardized surgical protocols. Therefore, its clinical application should be approached with caution. Future high-quality studies with extended follow-up are required to validate long-term efficacy and establish standardized clinical guidelines.

**Supplementary Information:**

The online version contains supplementary material available at 10.1186/s40729-025-00657-z.

## Introduction

With the continuous advancement of clinical techniques, dental implantation has become a common treatment for patients with tooth loss [1]. Aesthetic zone implantation, as a critical research area in modern oral implantology, is both a hotspot and a challenging domain. Tooth loss in this region not only compromises masticatory function but may also negatively impact patients’ social interactions and psychological well-being. As patients typically have high expectations for post-restoration aesthetic outcomes, achieving optimal outcomes in the aesthetic zone remains a significant clinical challenge [2, 3]. Conventional immediate implant placement (CIIP), which eliminates the need for socket healing, significantly shortens treatment duration and rapidly restores aesthetics, making it widely adopted in the aesthetic zone [4, 5]. However, studies indicated that even minimally traumatic extraction during CIIP cannot fully prevent alveolar bone remodeling, particularly resorption of the buccal bone plate [6–8]. This resorption primarily stems from the disruption of the bundle bone-periodontal ligament apparatus after tooth extraction. Deprived of vascular supply previously maintained by the periodontal ligament, the buccal bone plate becomes prone to progressive resorption and loss [9]. Preserving vascularization from the periodontal ligament may thus represent an effective strategy to mitigate peri-implant bone loss following CIIP.

Socket Shield Technique (SST) was first introduced by Hürzeler et al. [10] in 2010. This technique preserves a portion of the buccal root during tooth extraction, maintaining blood supply from the periodontal ligament to reduce physiological remodeling of surrounding tissues and achieve long-term preservation of the alveolar ridge contour. In their study, Hürzeler et al. [10] retained roots 1 mm coronal to the crest, which effectively prevented apical epithelial migration while preserving periodontal ligament tissue, thereby enhancing soft and hard tissue stability. Schwimer et al. [11] provided human histological evidence in a case report, demonstrating complete bone healing of the gap between the implant and buccal shield, achieving ideal osseointegration and further validating the feasibility of SST. However, postoperative complications such as osseointegration failure, internal and external root shield exposure had been reported [12–14]. To address these risks, modifications to SST had been proposed, focusing on the height and thickness of shield, and management of the gap between implant and shield [15–18]. For example, Gluckman et al. [15] positioned shield flush with the alveolar crest and prepared a 2 mm concave groove on the lingual aspect of the root. This design promoted soft tissue ingrowth into the gap, enhancing soft tissue sealing and reducing postoperative fragment exposure.

As SST becomes more widely applied in clinics, an increasing number of studies have compared its postoperative outcomes with those of CIIP. Although previous meta-analyses [19–22] had compared the postoperative clinical outcomes of SST and CIIP, their limitations such as outdated literature searches leading to insufficient study inclusion and language restrictions highlighted the necessity of updating the evidence with additional high-quality studies. This study comprehensively compared SST and CIIP in the aesthetic zone by synthesizing recent evidence from a larger pool of studies. By evaluating relevant outcomes, such as buccal bone loss and pink esthetic scores (PES), our findings aim to provide clinicians with updated and comprehensive evidence-based guidance for clinical decision-making.

## Materials and methods

This meta-analysis was performed according to the Preferred Reporting Items for Systematic Reviews and Meta-Analyses (PRISMA) [23]. The population, intervention, comparison, outcomes and study design (PICOS) framework [24] were used to guide the inclusion and exclusion of studies for the focused questions.

### Inclusion criteria

①Population: Patients who required immediate implant placement in the esthetic zone, defined as the region between and including the second premolars.

②Intervention: SST.

③Comparison: CIIP.

④Outcomes: Primary outcome: horizontal buccal bone loss; Secondary outcomes: vertical buccal bone loss, pink aesthetic score (PES), implant stability quotient (ISQ), and implant success rate. At least one outcome indicator was reported. Outcomes were further categorized into long-term outcomes (> 12 months) and short-term outcomes (≤ 12 months) based on follow-up duration.

⑤Study design: Randomized controlled trials (RCT) and non-randomized studies of interventions (NRSI).

### Exclusion criteria

①Studies for which the full text or required outcome data cannot be obtained.

②Patients with any disease, condition or medication that may compromise osseointegration or healing, such as osteoporosis, osteopenia, diabetes and bisphosphonates.

③Uncontrolled or untreated periodontitis, soft tissue recession or attachment loss on the tooth to be replaced.

④Less than 3 months follow-up.

### Search strategy

The literature search was independently conducted by two researchers. English databases included PubMed, Embase, Cochrane Library, and Web of Science, while Chinese databases comprised China National Knowledge Infrastructure (CNKI), Chinese Science and Technology Periodical Database (VIP), and Wanfang Database. The retrieval period was from database inception to October 9, 2024. A combination of keywords and free words and free-text keywords was used with logical operators to ensure comprehensive retrieval. The search terms included “immediate dental implant loading”, “socket shield technique”, “root membrane technique”, “partial extraction” and more. Details regarding the search strategy in these databases were summarized in Appendix Table 1.

**Table 1:** Basic characteristics of the included studies

Author, year	Study design	Age (year)	*Intervention* T / C	*Implants evaluated* T / C	Follow-up (month)	Implant distribution	Thickness of buccal shield	Height of buccal shield	Gap distance and bone grafting	Outcomes
Abd-Elrahman, 2020	RCT	21 ~ 39	SST/CII	20/20	6	Maxillary incisors and canines	1 mm	b	2 mm, none	①②③④⑤
Atef, 2021	RCT	36 ± 5.55	SST/CII	21/21	12	Maxillary premolars and anteriors	1 ~ 1.5 mm	b	2 mm, bone grafting only in C	①②③⑤
Bramanti, 2018	RCT	NA	SST/CII	20/20	36	Maxillary/Mandibular incisors and canines	NA	c	NA, bone grafting	②③⑤
Kumar, 2021	RCT	37	SST/CII	15/15	4	Maxillary incisors and canines	NA	b	NA, none	③
Santhanakrishnan, 2021	RCT	30.2 ± 8.1	SST/CII	25/25	6	Maxillary premolars and anteriors	NA	b	2 mm, both	①③
Sun, 2020	RCT	NA	SST/CII	15/15	24	Maxillary/Mandibular incisors and canines	1 mm	c	NA, bone grafting when the distance>1 mm	①②③⑤
Tiwari, 2020	RCT	18 ~ 30	SST/CII	8/8	12	Maxillary incisors and canines	NA	c	NA, none	①
Abdullah, 2022	RCT	29.4	SST/CII	23/23	12	Maxillary premolars and anteriors	NA	b	T: direct contact to each: C: bone grafting	①③④⑤
Venkatraman, 2023	RCT	18 ~ 45	SST/CII	11/11	12	Maxillary incisors and canines	1.5 ~ 2 mm	a	1 ~ 2 mm, none	③
Barakat, 2017	RCT	20 ~ 50	SST/CII	10/10	7	Maxillary incisors and canines	NA	c	NA, none	①②④⑤
Fattouh, 2018	RCT	NA	SST/CII	10/10	12	Maxillary incisors and canines	NA	a	NA, bone grafting only in C	②③⑤
Abdel-Raheim, 2019	RCT	20 ~ 35	SST/CII	10/10	6	Maxillary incisors and canines	NA	c	NA, none	①②④
Hana, 2020	RCT	28 ~ 65	SST/CII	20/20	12	Maxillary/Mandibular incisors and canines	2 mm	b	1–2 mm, none	③⑤
Zhang, 2020	RCT	37.0 ± 3.7	SST/CII	30/30	12	Maxillary incisors and canines	NA	NA	2 mm, both	①③⑤
Huang, 2024	RCT	21 ~ 59	SST/CII	61/61	12	Incisors and canines	1 mm	a	1 mm, both	①③⑤
Yan, 2019	RCT	37.8 ± 14.3	SST/CII	13/15	12	Maxillary incisors and canines	≥ 1.5 mm	b	T: bone grafting when >1 mm C: bone grafting	①③⑤
Hu, 2024	RCT	22 ~ 52	SST/CII	30/30	6	Maxillary incisors and canines	1 mm	a	T: bone grafting when gap ≥ 2 mm C: bone grafting	①④
Li, 2021	RCT	25 ~ 53	SST/CII	40/40	12	Maxillary incisors and canines	1 mm	a	2 mm, both	①③④⑤
Huo, 2022	RCT	18 ~ 35	SST/CII	20/20	6	Maxillary incisors and canines	1.5 ~ 2.0 mm	b	NA, only in C	①②③⑤
Li, 2023	RCT	27 ~ 52	SST/CII	62/68	6	Maxillary incisors and canines	1 mm	a	2 mm, both	①④
Liu, 2022	RCT	60 ~ 79	SST/CII	29/31	12	Maxillary incisors and canines	1 mm	a	2 mm, both	①④
Zhou, 2023	RCT	22 ~ 51	SST/CII	49/49	24	Incisors and canines	1 mm	NA	2 mm, both	①③⑤
Peng, 2023	NRSI	≥ 18	SST/CII	30/30	12	Maxillary incisors and canines	1 mm	a	NA, both	①③⑤
Xu, 2019	NRSI	25 ~ 53	SST/CII	12/12	12	Maxillary incisors and canines	1 mm	a	2 mm, both	①③⑤
Li, 2022	NRSI	≥ 18	SST/CII	18/18	12	Maxillary incisors and canines	1 ~ 1.5 mm	b, c	T: bone grafting when gap > 1 mm C: bone grafting	①③⑤
Yao, 2022	NRSI	19 ~ 51	SST/CII	27/30	18	Maxillary incisors and canines	1 mm	b	T: 1 ~ 2 mm; C: 2 ~ 3 mm, both	①③⑤
Li, 2024	NRSI	RCT	SST/CII	18/18	60	Maxillary incisors and canines	NA	c	NA, both	①③

RCT: randomized controlled trial; NRSI: non-randomised studies of the effects of interventions; T: test group; C: control group; SST: socket Shield Technique; CIIP: conventional immediate implant placement; a: above the level of crestal bone; b: at the level of crestal bone; c: below the level of crestal bone; ①: horizontal buccal bone loss; ②: vertical buccal bone loss; ③: pink aesthetic score; ④: implant stability quotient; ⑤: implant success rate; gap distance: the distance of the gap between the implant and buccal shield/buccal bone; NA: not available. In the line of “Gap distance and bone grafting”, “both” indicated bone grafting was performed in both control group and test group; “none” indicated no bone grafting was performed in either group; “when the distance >? mm” indicated that bone grafting was only performed when the gap distance was greater than ? mm

### Study selection and data extraction

All retrieved Chinese and English literatures were imported into EndNote X9 for literature screening and management. Duplicates were first removed using the software’s deduplication function. Subsequently, two researchers independently screened the titles and abstracts of the remaining studies based on predefined inclusion and exclusion criteria to exclude irrelevant studies. Full texts of the preliminarily eligible studies were further assessed for eligibility. After screening, cross-checking was performed between the two researchers to ensure consistency. In cases where disagreements arose, discussions were held to reach a consensus. If a consensus could not be reached, a third researcher was consulted to make the final decision. The data from the included studies were extracted independently by two researchers with pre-designed data extraction forms. The following information was extracted: author names, publication year, study design, follow-up duration, implant sample size, age of patients, interventions, implant location, the thickness and height of buccal shield, outcome indicators, the distance of the gap between the implant and buccal bone plate, and bone grafting.

### Quality assessment and risk of bias

The risks of bias assessment of the included studies were assessed by two reviewers independently. For RCTs, the revised version of Cochrane Risk of Bias Tool (RoB 2.0) [25] was used to assess the risk of bias. Five domains were assessed mainly, including bias from the randomization process, deviation from intended interventions, missing outcome data, measurement of outcomes, and selection of results. Responses to signaling questions within each domain were categorized as “Yes (Y),” “Probably Yes (PY),” “Probably No (PN),” “No (N),” or “No Information (NI).” The tool automatically generated an overall risk-of-bias judgment for each study, classifying results as “High risk,” “Some concerns” and “Low risk” lastly. For NRSIs, risk of bias in non-randomised studies of interventions (ROBINS-I) [26] was applied as a tool to evaluate the potential risk of bias according to following seven domains, including bias due to confounding, bias in selection of participants into the study, bias in classification of interventions, bias due to deviations from intended interventions, bias due to missing data, bias in measurement of outcomes and bias in selection of the reported result. Each domain was evaluated based on predefined criteria, with final judgments categorized as “Low risk,” “Moderate risk,” “Serious risk” and “Critical risk.” The “No information” category should be used only when insufficient data were reported to permit a judgement. Two reviewers independently conducted the assessments, followed by cross-checking. If there was any disagreement, a third reviewer could assist with the final decision-making after reading the original studies.

### Statistical analysis

Statistical analysis was performed by RevMan 5.4 software. Continuous data outcomes, such as horizontal and vertical buccal bone loss, were expressed as mean difference (MD) with 95% confidence interval (CI). Dichotomous data outcomes, such as implant success rate, were presented as risk ratio (RR) with 95% CI. The statistical heterogeneity among studies in the meta-analysis was assessed using the I^2^ test. A fixed-effects model was selected when I^2^ ≤ 50% (indicating low heterogeneity), while a random-effects model was applied when I^2^ >50% (indicating significant heterogeneity). Statistical significance was defined as *P* < 0.05. Meta-analyses were conducted based on the maximum follow-up data for each outcome indicator in the long-term and short-term. For the primary outcome, subgroup analyses were further stratified by the thickness and height of buccal shield and bone grafting. Studies employing inconsistent treatment protocols between groups, such as the selective use of bone grafting in the gap, were excluded before conducting the subgroup analyses mentioned above. Subgroup analyses stratified by the publication language of the included studies were also performed for all outcome indicators to evaluate potential language-related biases. To ensure the stability and reliability of the results, sensitivity analyses were conducted by excluding NRSIs and studies with inconsistent interventions between or within groups for verification. Finally, funnel plot analysis was used to assess whether there was any publication bias among the included studies.

## Results

### Result of literature search

**Fig. 1:**
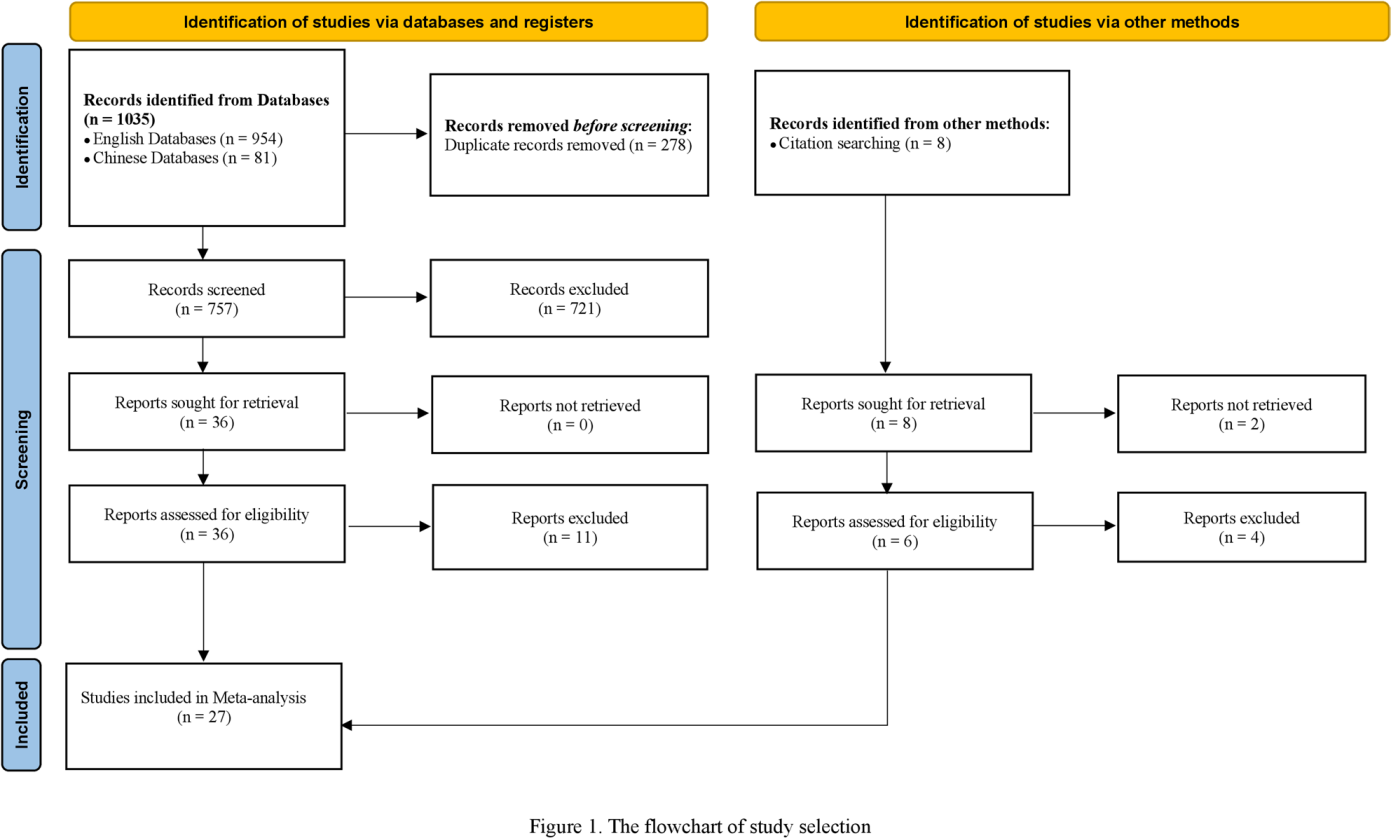
The flowchart of study selection

The literature screening process and results were shown in Fig. 1. Initially, a total of 1043 records were identified as potentially eligible studies from seven databases and other methods. After removing 278 duplicates, preliminary screening of titles and abstracts led to the exclusion of 721 studies. Subsequent full-text review and stepwise exclusion were performed in a total of 42 studies, ultimately resulting in the inclusion of 27 studies [27–53].

### Basic characteristics of the included studies

The basic characteristics of all the included studies were shown in Table 1. A total of 27 studies [27–53] were included in this study, with 13 published in Chinese [40–52] and 14 in English [27–39, 53]. Among these, 22 were RCTs [27–39] and 5 were NRSIs [49–53]. A total of 1307 implants were analyzed, including 647 implants in the SST group and 660 implants in the CIIP group. All studies reported a follow-up duration of at least 4 months. Regarding implant regions, 3 studies [29, 32, 39] involved both maxillary and mandibular anterior and premolar regions, 2 studies [41, 48] focused exclusively on the anterior region, and the remaining studies were conducted in the maxillary aesthetic zone. All studies reporting root thickness specified a minimum thickness of ≥ 1 mm. For the height of root relative to the level of crestal bone, 9 studies [35, 37, 41, 43, 44, 46, 47, 49, 50] positioned shield above the level of crestal bone, 9 studies [27, 28, 30, 31, 34, 39, 42, 45, 52] aligned shield parallel to the level of crestal bone, and 6 studies [29, 32, 33, 36, 38, 53] placed shield below the level of crestal bone. 2 studies [40, 48] did not report the height of buccal shield, and 1 study [51] described root below the crest in the control group but parallel in the SST group. For the gap between implants and labial structures (buccal shield in the SST group or buccal bone plate in the CIIP group), most studies maintained a width of ≥ 1 mm (typically 2 mm). Bone grafting was performed in both groups in most cases, though some studies [28, 32, 34, 37, 42, 43, 45, 51] performed only in the CIIP group or only when the SST group gap exceeded a specific threshold. Notably, in the study published by Abdullah et al. [34], implants in the SST group directly contacted the root fragment, while bone grafting was performed in the CIIP group.

### Risk of bias assessment

**Fig. 2:**
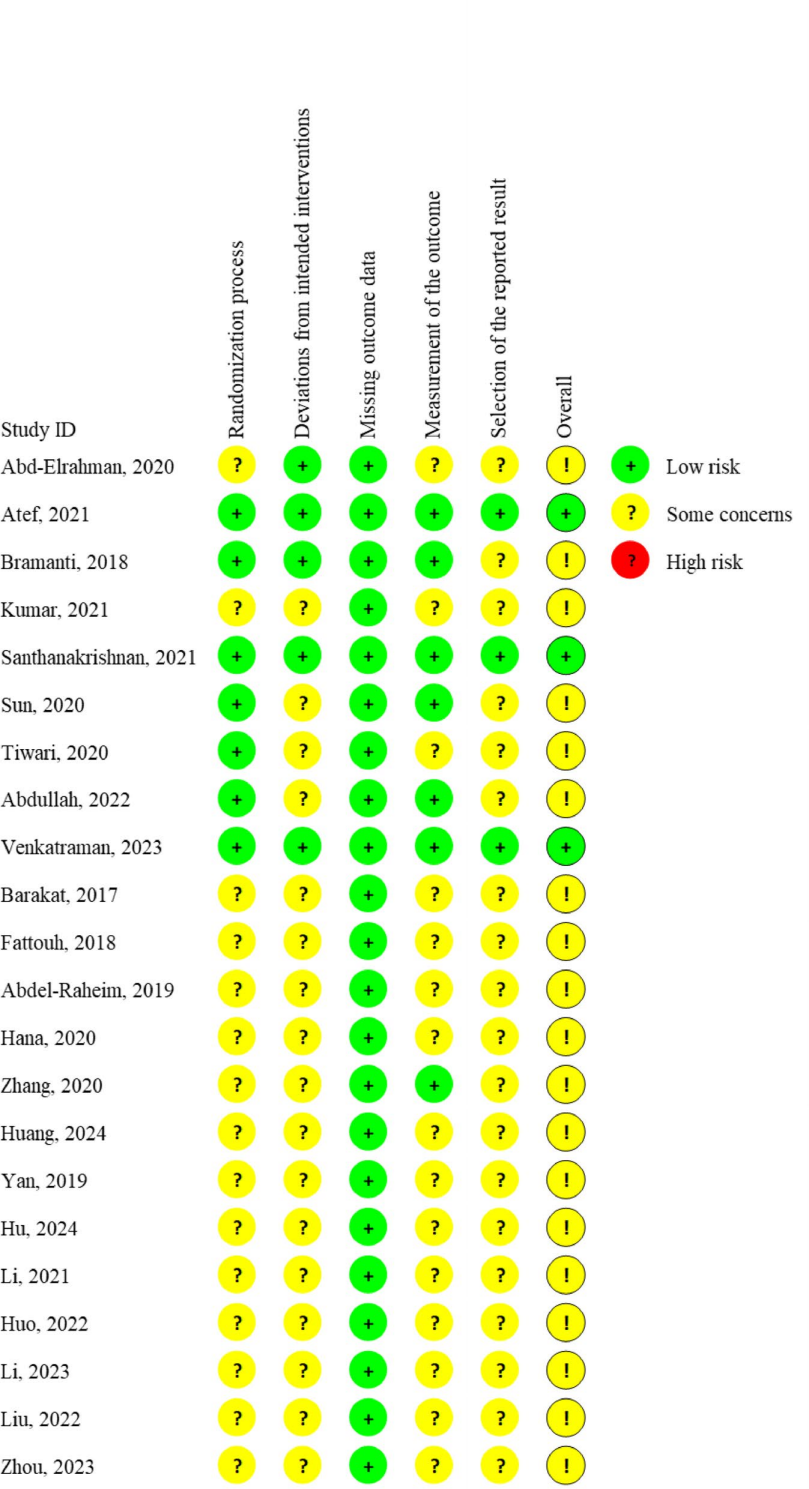
Risk of bias assessment of the included randomized controlled trials

Among the included RCTs [27–39], 3 studies [28, 31, 35] received an assessment of “Low”, while the remaining were classified as “Some concerns.” All studies mentioned random allocation, but most failed to provide detailed descriptions of the randomization method. Due to the specificity of the interventions, all clinicians performing the procedures were unavoidably aware of the techniques employed, making blinding of the operators impossible. However, no deviations from intended interventions caused by study settings or clinicals factors were reported in most studies. Regarding “Missing outcome data,” all studies were judged as “Low risk.” Additionally, only 3 studies [28, 31, 35] allowed comparison between reported outcomes and pre-specified research protocols. For the included NRSIs [49–53], 3 studies [49, 50, 53] were rated as “Moderate risk” and 2 [51, 52] as “High risk.” In terms of “Bias in selection of participants into the study,” 1 study [52] was deemed as “High risk” due to inconsistent exclusion criteria between the control and test groups. 1 study [51] exhibited discrepancies in gap management between the test and control groups, potentially affecting primary outcomes, and was thus rated as “High risk” for “Bias due to confounding” and “Bias in classification of interventions.” Detailed risk of bias assessments was presented in Fig. 2; Table 2.

**Table 2:** Risk of bias assessment of the included non-randomized studies of the effects of interventions

Author, year	D1	D2	D3	D4	D5	D6	D7	Overall bias
Peng, 2023	Moderate	Moderate	Moderate	Low	Low	Moderate	Low	Moderate
Xu, 2019	Moderate	Moderate	Low	Low	Low	Moderate	Low	Moderate
Li, 2022	High	Low	High	Moderate	Low	Moderate	Low	High
Yao, 2022	Moderate	High	Moderate	Low	Low	Low	Low	High
Li, 2024	Moderate	Low	Low	Low	Low	Low	Low	Moderate

D1: Bias due to confounding; D2: Bias in selection of participants into the study; D3: Bias in classification of interventions; D4: Bias due to deviations from intended interventions; D5: Bias due to missing data; D6: Bias in measurement of outcomes; D7: Bias in selection of the reported result

### Data synthesis

#### Primary outcomes

**Fig. 3:**
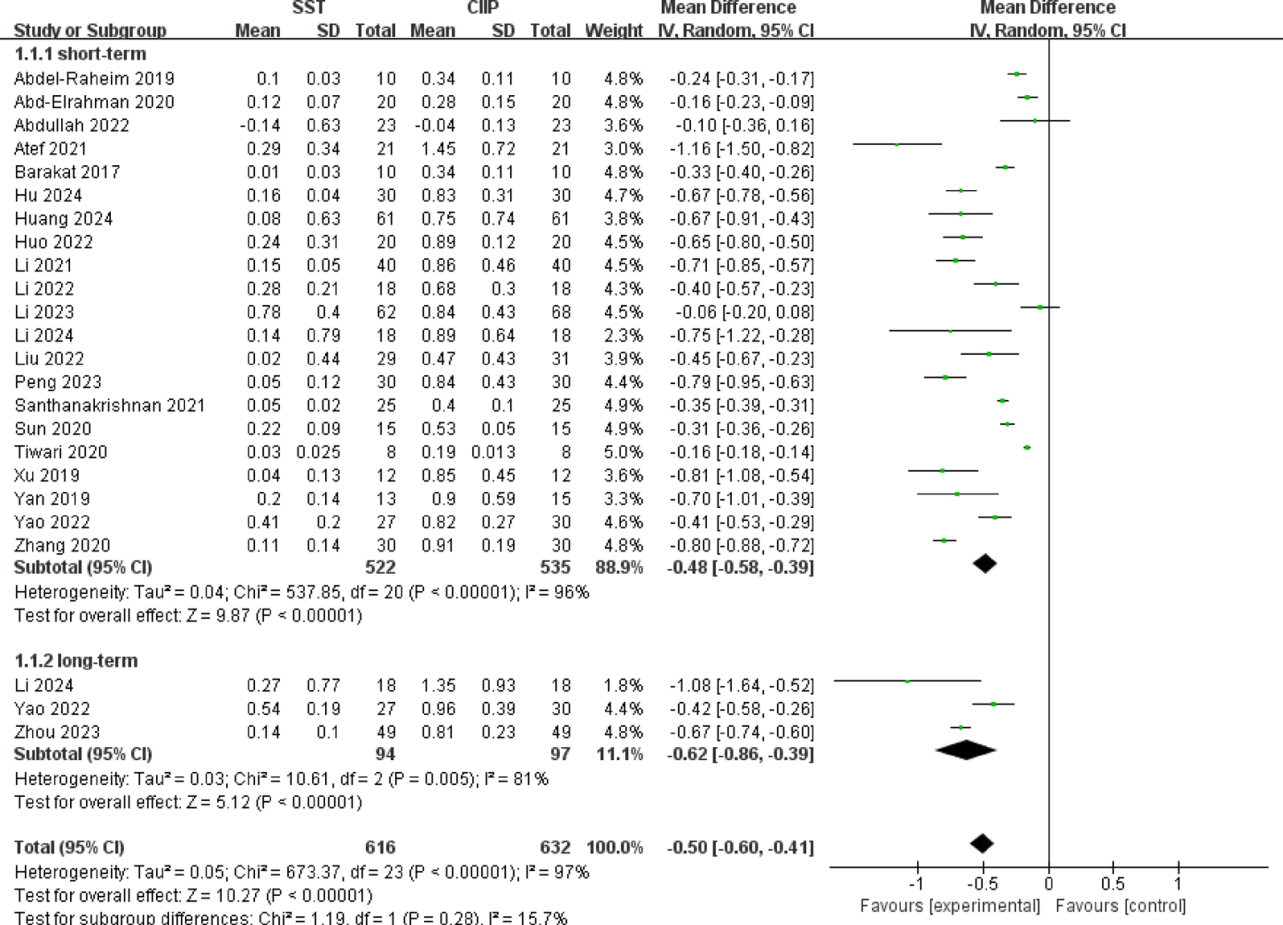
Forest plot for horizontal buccal bone loss

For horizontal buccal bone loss, a total of 22 studies [27, 28, 31–34, 36, 38, 40–53] reported horizontal buccal bone loss, involving 1155 implants. As shown in Fig. 3, meta-analysis revealed significant heterogeneity among studies (*P* < 0.00001, I^2^ = 97%), prompting the use of a random-effects model. Overall, SST demonstrated statistically significant superiority over CIIP in reducing horizontal buccal bone loss [MD = −0.50, 95%CI (−0.60, −0.41), I^2^ = 97%]. Subgroup analyses stratified by follow-up duration further revealed consistent benefits of SST in both short-term [MD = −0.48, 95%CI (−0.58, −0.39), I^2^ = 96%] and long-term [MD = −0.62, 95%CI (−0.86, −0.39), I^2^ = 81%], with statistically significant differences favoring SST in all analyses.

#### Secondary outcomes

**Fig. 4:**
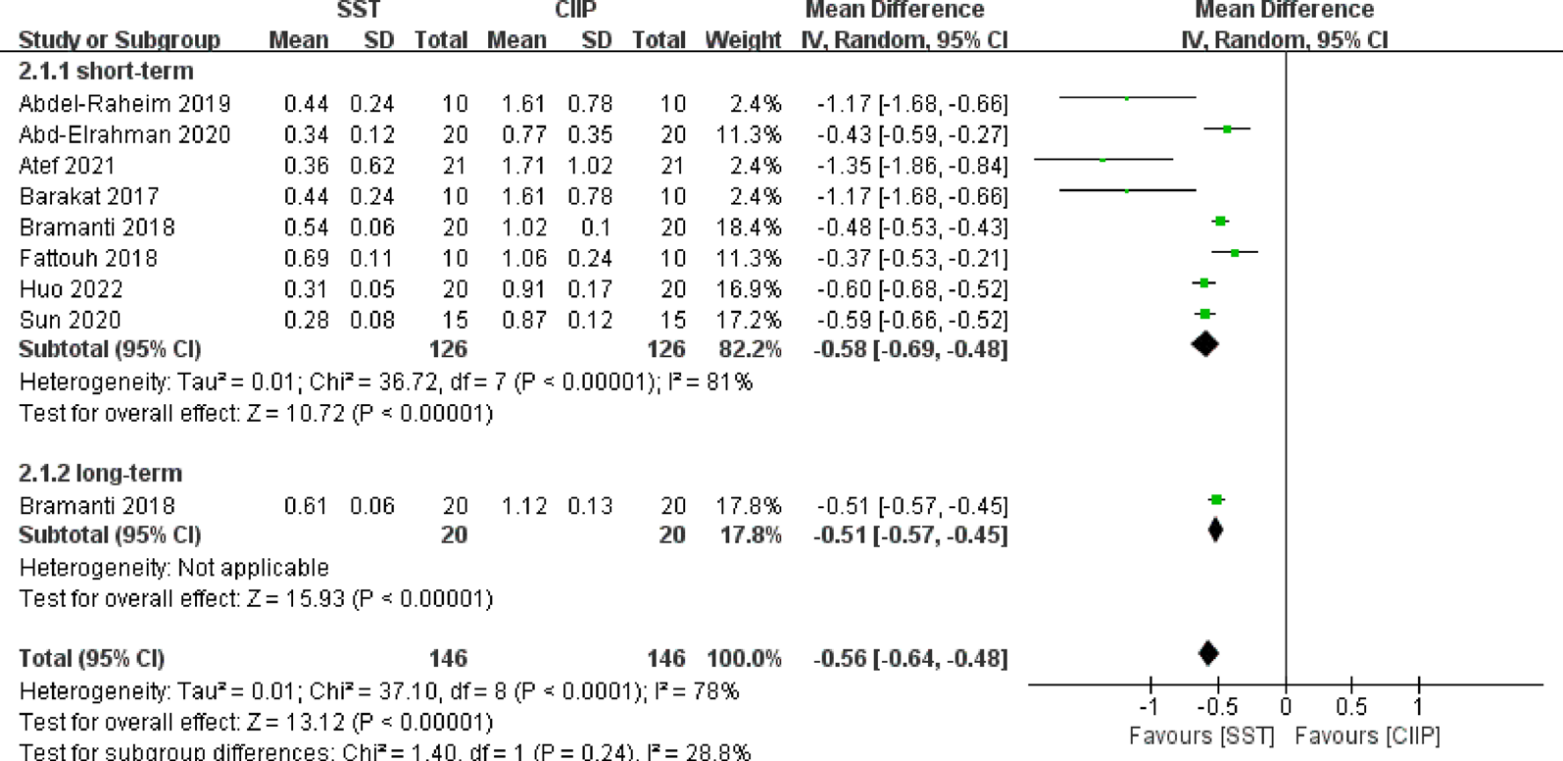
Forest plot for vertical buccal bone loss

For vertical buccal bone loss, a total of 8 studies [27–29, 32, 36–38, 45] involving 252 implants reported vertical buccal bone loss. Heterogeneity analysis indicated substantial between-study variation (*P* < 0.0001, I^2^ = 78%), necessitating the use of a random-effects model. Meta-analysis demonstrated that SST significantly outperformed CIIP in reducing vertical buccal bone loss [MD = −0.56, 95%CI (−0.64, −0.48), I^2^ = 78%]. In the short-term, SST showed a statistically significant reduction in vertical buccal bone loss compared to CIIP [MD = −0.58, 95%CI (−0.69, −0.48), I^2^ = 81%]. For long-term, only one study [29] reported vertical buccal bone loss, with result consistent with the overall analysis [MD = −0.51, 95%CI (−0.57, −0.45)]. The results were summarized in Fig. 4.

**Fig. 5:**
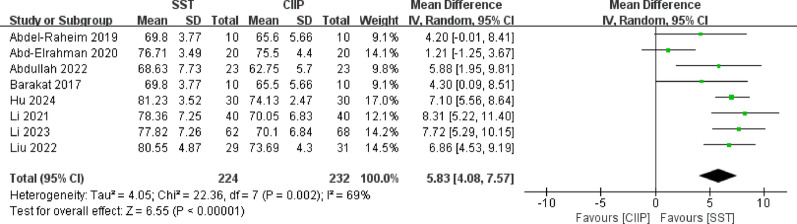
Forest plot for implant stability quotient

Regarding implant stability quotient, a total of 8 studies [27, 34, 36, 38, 43, 44, 46, 47] involving 456 implants reported ISQ, with all studies having a follow-up duration not exceeding 12 months. Heterogeneity analysis indicated significant heterogeneity among the studies (*P* = 0.002, I^2^ = 69%), prompting the use of a random-effects model. Meta-analysis demonstrated that SST was significantly superior to CIIP in improving ISQ [MD = 5.83, 95%CI (4.08, 7.57), I^2^ = 69%], as illustrated in Fig. 5.

**Fig. 6:**
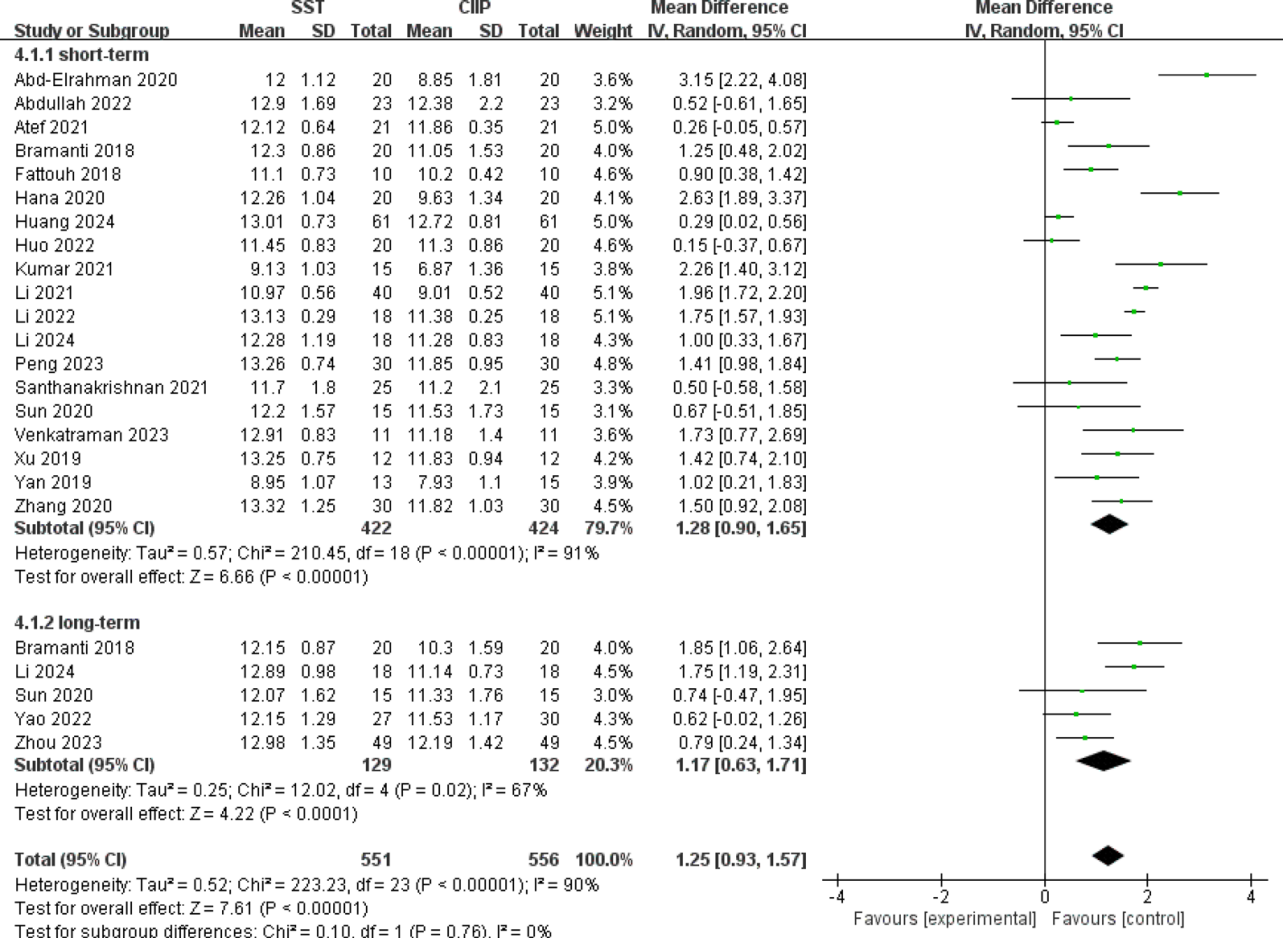
Forest plot for pink aesthetic score

For pink aesthetic score, a total of 21 studies [27–32, 34, 35, 37, 39–42, 44, 45, 48–53] involving 1001 implants reported PES. Significant heterogeneity was observed among the studies (*P* < 0.00001, I^2^ = 90%), leading to the use of a random-effects model for data analysis. Meta-analysis demonstrated that SST significantly improved PES compared to CIIP [MD = 1.25, 95%CI (0.93, 1.57), I^2^ = 90%]. Subgroup analyses stratified by follow-up duration further revealed consistent results, with both short-term [MD = 1.28, 95%CI (0.90, 1.65), I^2^ = 91%] and long-term [MD = 1.17, 95%CI (0.63, 1.71), I^2^ = 67%] aligning closely with the main result. These results were summarized in Fig. 6.

**Fig. 7:**
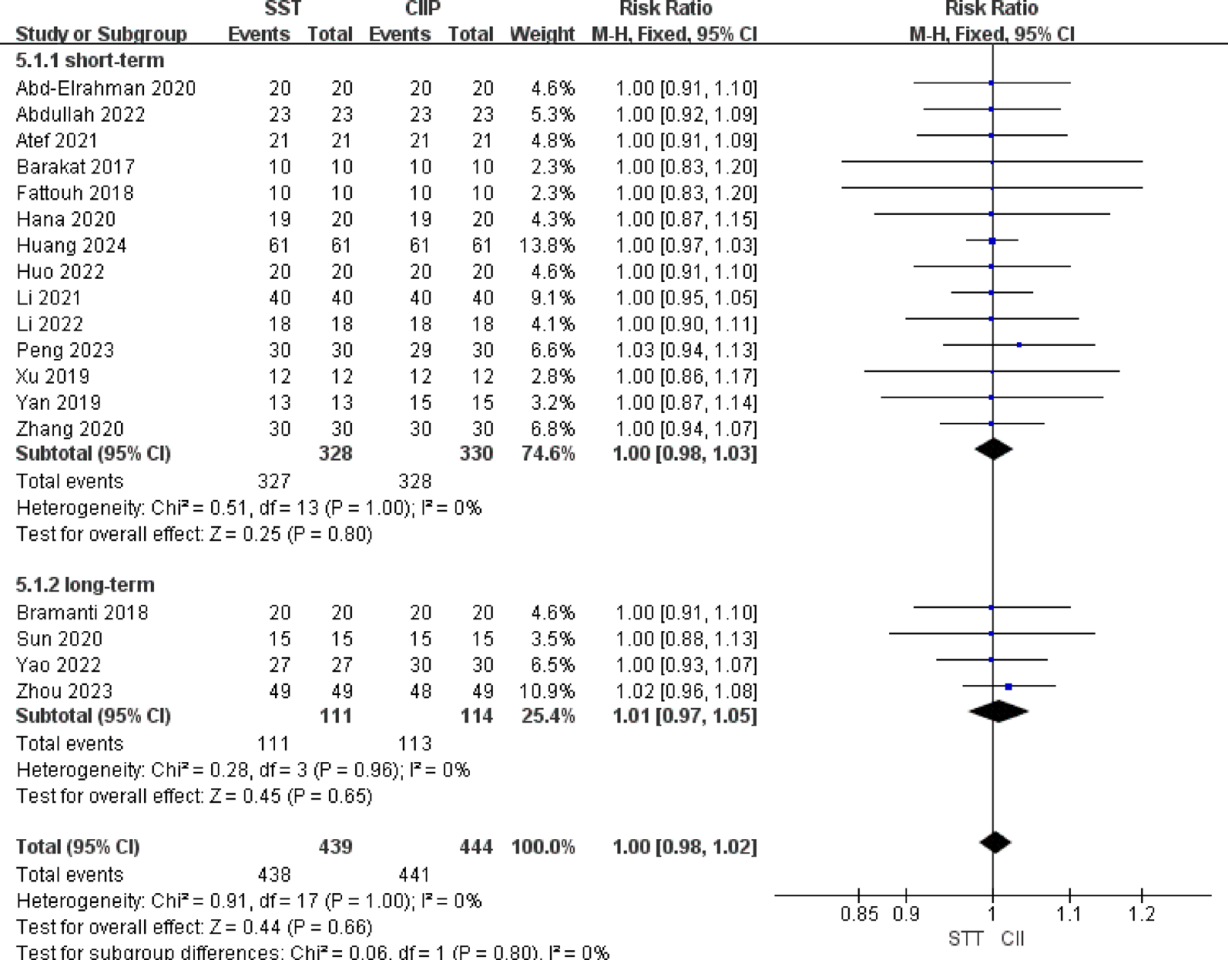
Forest plot for implant success rate

With regard to implant success rate, a total of 18 studies [27–29, 32, 34, 36, 37, 39–42, 44, 45, 48–52] involving 883 implants reported implant success rate. Heterogeneity analysis revealed no significant between-study heterogeneity (*P* = 1.00, I^2^ = 0%), and a fixed-effects model was therefore applied. Overall, no statistically significant difference in implant success rate was observed between the SST and CIIP groups [RR = 1.00, 95%CI (0.98, 1.02), I^2^ = 0%]. The SST group had 1 implant failure in the short term, while the CIIP group reported 2 short-term failures and 1 additional long-term failure. However, neither the short-term [RR = 1.00, 95%CI (0.98, 1.03), I^2^ = 0%] nor long-term [RR = 1.01, 95%CI (0.97, 1.05), I^2^ = 0%] follow-up analyses showed statistically significant differences between the groups. The results were illustrated in Fig. 7.

#### Subgroup analyses

Subgroup analyses were performed for primary outcomes based on height of buccal shield, thickness of buccal shield, and bone grafting. Additionally, all outcomes were stratified by publication language into Chinese and English subgroups. All results were presented in Tables 3 and 4.

**Table 3:** Results of subgroup analyses of primary outcome (horizontal buccal bone loss)

Factors	Subgroups	Included studies	*Implants* T/C	MD [95%CI]	*P*	I^2^	*P* (I^2^)	Effect model
height of buccal shield	a	7	264/272	−0.59 [−0.80, −0.38]	< 0.00001	91%	< 0.00001	random
	b	7	149/154	−0.46 [−0.62, −0.30]	< 0.00001	92%	< 0.00001	random
	c	5	61/61	−0.28 [−0.38, −0.18]	< 0.00001	93%	< 0.00001	random
thickness of buccal shield	≤ 1.5 mm	12	396/407	−0.55 [−0.71, −0.40]	< 0.00001	95%	< 0.00001	random
	>1.5 mm	2	33/35	−0.66 [−0.79, −0.53]	< 0.00001	0%	0.77	fixed
bone grafting	Yes	11	383/394	−0.59 [−0.75, −0.44]	< 0.00001	95%	< 0.00001	random
	No	4	48/48	−0.22 [−0.30, −0.14]	< 0.0001	88%	< 0.00001	random

This table summarized the data from the subgroup analyses of the primary outcome, which included a total of 3 factors that may have brought about an impact on the results; a: above the level of crestal bone; b: at the level of crestal bone; c: below the level of crestal bone; T: test group; C: control group; MD: mean difference (MD less than 0 indicated that the result of test group was better than control group); CIs: confidence intervals; *P*<0.05 indicated that difference between test group and control group was statistically significant

**Table 4:** Results of subgroup analyses based on publication language

Outcomes	Subgroups	Included studies	*Implants* T/C	MD / RR [95%CI]	*P*	I^2^	*P* (I^2^)	Effect model
horizontal buccal bone loss	English	9	150/150	−0.31 [−0.40, −0.22]	< 0.00001	94%	< 0.00001	random
	Chinese	13	421/434	−0.60 [−0.71, −0.48]	< 0.00001	88%	< 0.00001	random
pink aesthetic score	English	11	198/198	1.49 [0.87, 2.10]	< 0.00001	88%	< 0.00001	random
	Chinese	10	300/305	1.10 [0.64, 1.57]	< 0.00001	93%	< 0.00001	random
implant stability quotient	English	4	63/63	3.09 [1.39, 4.80]	0.0004	37%	0.19	fixed
	Chinese	4	161/169	7.31 [6.25, 8.39]	< 0.00001	0%	0.87	fixed
implant success rate	English	8	139/139	1.00 [0.96, 1.04]	1.00	0%	1.00	fixed
	Chinese	10	300/305	1.01 [0.98, 1.03]	0.56	0%	1.00	fixed

This subgroup analysis stratified each outcome data by publication language (Chinese and English), with analyses performed using data from the longest follow-up duration in each study. T: test group; C: control group; MD: mean difference; RR: risk ratio CIs: confidence intervals; *P*<0.05 indicated that difference between test group and control group was statistically significant

For height of buccal shield, a total of 7 studies [41, 43, 44, 46, 47, 49, 50] involving 536 implants reported that the height of shield was above the level of crestal bone. Meta-analysis using a random-effects model (*P* < 0.00001, I^2^ = 91%) demonstrated that SST significantly reduced horizontal buccal bone loss compared to CIIP [MD = −0.59, 95%CI (−0.80, −0.38), *P* < 0.00001]. In 7 studies [27, 28, 31, 34, 42, 45, 52] with 303 implants where the height of shield was maintained at the crestal bone level, random-effects meta-analysis (*P* < 0.00001, I^2^ = 92%) also showed significant advantages of SST over CIIP [MD = −0.46, 95%CI (−0.62, −0.30), *P* < 0.00001]. Lastly, 5 studies [32, 33, 36, 38, 53] involving 122 implants showed that the height of shield was lower than crestal bone level, and meta-analysis under a random-effects model (*P* < 0.00001, I^2^ = 93%) revealed that SST remained superior to CIIP in reducing horizontal buccal bone loss [MD = −0.28, 95%CI (−0.38, −0.18), *P* < 0.00001].

As to thickness of buccal shield, a total of 12 studies [27, 28, 32, 41, 43, 44, 46–50, 52] with the thickness of shield ≤ 1.5 mm, involving 803 implants, were included. Meta-analysis using a random-effects model (*P* < 0.00001, I^2^ = 95%) showed that the SST significantly reduced horizontal buccal bone loss in implants compared to CIIP [MD = −0.55, 95%CI (−0.71, −0.40), *P* < 0.00001]. Additionally, 2 studies [42, 45] with shield’s thickness >1.5 mm, comprising 68 implants, were also analyzed. A fixed-effects model analysis (*P* = 0.77, I^2^ = 0%) indicated that SST also significantly reduced horizontal buccal bone loss compared to CIIP [MD = −0.66, 95%CI (−0.79, −0.53), *P* < 0.00001].

Regarding bone grafting, a total of 11 studies [31, 40, 41, 44, 46–50, 52, 53] involving 777 implants performed bone grafting in the implant-root gap. A random-effects model analysis (*P* < 0.00001, I^2^ = 95%) demonstrated that SST significantly reduced horizontal buccal bone loss compared to CIIP [MD = −0.59, 95%CI (−0.75, −0.44), *P* < 0.00001]. In contrast, 4 studies [27, 33, 36, 38] with 96 implants did not perform bone grafting in the gap. Meta-analysis using a random-effects model analysis (*P* < 0.00001, I^2^ = 88%) also showed the superiority of SST over CIIP in reducing horizontal buccal bone loss [MD = −0.22, 95%CI (−0.30, −0.14), *P* < 0.0001].

For publication language, the findings demonstrated that compared to CIIP, SST maintained significant advantages in reducing horizontal buccal bone loss, improving ISQ, and enhancing PES. However, no statistically significant difference was observed in implant success rates between SST and CIIP. Besides, heterogeneity showed a significant reduction for ISQ but remained unchanged for other outcomes. Regarding vertical buccal bone loss, subgroup analysis was not performed due to the inclusion of only one study published in Chinese.

#### Sensitivity analysis

The results of the sensitivity analysis were presented in Appendix Figs. 1, 2, 3, 4, 5, 6, 7 and 8. After excluding 5 NRSI [49–53], 3 outcomes were affected, including horizontal buccal bone loss, PES, and implant success rate. Furthermore, following the exclusion of 8 studies [28, 32, 34, 37, 42, 43, 45, 51] with inconsistent bone grafting protocols between or within groups, the data analyses for all outcome measures were affected. Overall, the sensitivity analyses revealed results consistent with the primary analyses, demonstrating that the findings remained relatively stable and reliable.

### Assessment of publication bias

The number of studies included was greater than 10 in 3 outcomes, and funnel plots were constructed for these outcomes. The funnel plots revealed a nearly symmetrical distribution of studies, suggesting that the possibility of significant publication bias was low. The details were presented in Appendix Fig. 4.

## Discussion

SST had provided a novel alternative for immediate implantation in the aesthetic zone. However, whether SST achieves more favorable clinical outcomes compared to CIIP remains debated. This meta-analysis included 27 studies [27–53], involving 1307 implants, to systematically compare the postoperative clinical effects of CIIP and SST across five outcome indicators: horizontal and vertical buccal bone loss, PES, ISQ, and implant success rate. Subgroup analyses were further conducted based on critical surgical variables aiming to provide clinicians with updated and comprehensive evidence-based guidance.

The finding of this meta-analysis demonstrated that SST showed significant advantages over CIIP in reducing both horizontal and vertical buccal bone loss. Previously published meta-analyses [19, 20] also concluded that SST significantly reduced labial bone loss, aligning with our results. However, caution is warranted when interpreting long-term bone loss differences, as few studies included follow-up durations exceeding 12 months for these outcomes. Notably, only 2 studies [34, 46] failed to observe significant reductions in horizontal buccal bone loss with SST. Upon reviewing the original articles, Abdullah et al. [34] performed bone grafting in the implant-labial bone plate gap for the CIIP group but omitted grafting in the SST group, where implants directly contacted root fragments. This methodological discrepancy may explain the divergent outcomes. Given the substantial heterogeneity observed in the primary outcome, subgroup analyses were conducted to explore potential influencing factors within the height and thickness of buccal shield, and bone grafting in the gap between implant and shield.

The management of shield during SST is critical to clinical success, with their thickness and height significantly influencing postoperative outcomes. Regarding buccal shield positioning, Hürzeler et al. [10] initially recommended retaining the root 1 mm above the crest to prevent apical epithelial migration and preserve periodontal ligament integrity for soft tissue stability. Mitsias et al. [54] similarly suggested positioning the root 0.5–1 mm above the crest to support buccal soft tissues while maintaining connective tissue for biological sealing. In contrast, some scholars argued that excessively coronal positioning increases risks of exposure and fracture, advocating for the root alignment with or apical to the crest to minimize the risk of exposure [15, 16]。In this study, root fragments were categorized into three subgroups based on their vertical positioning relative to the alveolar crest: below, above, or aligned with the crest. Subgroup analysis of horizontal buccal bone loss revealed that SST consistently demonstrated statistically significant reductions in bone loss compared to CIIP in all groups. The thickness of shield similarly impacted the outcomes of SST. Gluckman et al. [55] suggested that root fragments thinner than 1.5 mm compromised shield stability, and the preparation of such thin fragments presented significant technical challenges that may undermine alveolar ridge preservation. Relevant animal studies had shown that when the thickness of shield ranged from 0.5 to 1.5 mm, thicker thicknesses result in less buccal bone loss; when the thickness exceeded 2 mm, the possibility of root shield displacement may increase [56, 57]. In this study, subgroup analyses were conducted based on whether the thickness of shield exceeded 1.5 mm. However, the results of the subgroup analysis showed that regardless of whether buccal shield were ≤ 1.5 mm or >1.5 mm in thickness, SST was significantly more effective than CIIP in preventing horizontal loss of the buccal bone. Although both the height and thickness of buccal shield may influence the outcomes following SST, due to the limited number of available studies, more high-quality studies are still needed to further verify its potential effects.

Regarding whether to retain the gap between the implant and the root and whether bone grafting is needed to fill the gap, there is still controversy. Different gap management methods may affect the marginal bone level of the implant [10, 55, 58]. Some studies had shown that the more distally placed the implant, the more likely it is to cause increased loss of the labial bone, and even potentially determine the degree of recession of the peri-implant tissues [59, 60]. Initially, Hürzeler et al. [10] did not leave a gap between the implant and the root, and Mitsias et al. [54] also believed that making the shield adjacent to the implant can provide it with better positioning and stability. Zuhr et al. [61] further pointed out that when the root membrane contacted the implant, it can be securely fixed by the threads of the implant, effectively inhibiting the vertical displacement of the root membrane during bone remodeling. However, some clinical and histological studies suggested that retaining the gap between the implant and the buccal shield, on the one hand, benefited the growth of new bone in the gap during dental implant healing stages, which in turn enhanced stability, and on the other hand, simultaneously increased peri-implant soft tissue thickness for improved biological sealing. Regarding whether to graft materials in this gap, Siormpas et al. [58] argued that no grafting materials were necessary, while Gluckman et al. [62, 63] recommended filling any existing gap with bone grafting material. Botticelli et al. [64] proposed that no bone grafting was needed when the skip gap is between 0.5 and 1 mm, and when the gap was greater than 1 mm, filling with bone grafting material is necessary to prevent infection. Among all the studies included reporting on the gap, most of them exceeded 1 mm. Only the study published by Abdullah et al. [34] specifically stated that the implant was placed in direct contact with the shield. The results for horizontal bone loss in the SST group showed no significant advantage compared to the CIIP group, which contradicted findings from other studies. This discrepancy might be because direct contact between implant and shield positioned the implant more labially, potentially leading to greater bone plate loss. However, it was noteworthy that bone grafting was performed only in the CIIP group in that study, introducing a confounding factor. Furthermore, a subgroup analysis of the included studies reporting horizontal bone loss based on whether bone grafting was performed also conducted, and the result indicated that the SST group demonstrated significantly less horizontal buccal bone loss than the CIIP group, regardless of whether bone grafting was performed. For the influence of different gap distances and treatment methods on the peri-implant bone tissue following SST, more high-quality, well-designed experiments are still needed to further verify.

This study evaluated the aesthetic differences between the two groups based on the PES proposed by Fürhauser et al. [65], considering 7 main parameters: the mesial and distal papilla levels, the soft tissues contour, level, texture, and color, and the alveolar process deficiencies. The results indicated that SST demonstrated a significant advantage over CIIP in improving PES, both in the long term and short term, which is consistent with the findings of other published meta-analyses [19, 20, 66]. This advantage may be attributed to the preservation of the root fragment, which effectively maintained the corresponding physiological structure of the buccal bone. Consequently, the peri-implant soft tissue had stable bony support, thereby preserving volume stability. Regarding ISQ, the result showed that compared to CIIP, SST achieved significantly higher ISQ values than CIIP postoperatively. However, since all studies had a follow-up period of no more than 12 months, whether long-term ISQ differed remained unclear and required further investigation.

In terms of implant success rates, the results showed that SST failed to demonstrate a significant advantage compared to CIIP. Among all the included studies, only 3 studies [39, 48, 49] reported cases of implant failure, involving a total of 4 implants. Among them, only Hana et al. [39] reported a failure case within the SST group. Their study noted that despite the excellent bone stability around the implant and good level of marginal bone observed in the successful cases of the SST group, there were still issues with the exposure of the shield both inside and outside as well as insufficient soft tissue. The possible reason for the fragment exposure in this study was likely related to the preparation of the fragment being positioned 1 mm above the crest bone. Overall, while there were failure cases in the SST group, the success rate in the SST group was comparable to that of the CIIP group. However, due to the limited evidence, there was still a need for more research to further verify if there was any difference in long-term success rates.

Although SST demonstrated promising outcomes such as implant success rate, it still faced challenges from complications like the exposure of the shield. As illustrated by the failure case of SST reported by Hana et al. [39], such complications had the possibility of affecting clinical outcomes by compromising aesthetics, patient satisfaction, and prosthetic success. Consequently, systematic evaluation of postoperative complications remained essential. Therefore, a thorough evaluation of postoperative complications of SST remained essential. Ogawa et al. [67] found that internal shield exposure as the most common complication (46%), followed by failure of osseointegration (19%), external shield exposure (15%), shield mobility and infection (12%), shield migration (4%), and apical root resorption (4%). Among the included studies, Abd-Elrahman et al. [27] reported a case of internal shield exposure, Hana et al. [39] reported two cases of complications with one being an internal and the other an external shield exposure. Besides, Hu et al. [43] and Li et al. [46] each reported one case of infection in their respective studies. In the study of Li et al. [53], one shield was extracted 1 year after the final restoration. Fortunately, most complications were non-progressive, resulting in no notable bone defects or aesthetic recession and thereby avoiding the need for surgical intervention. Gluckman et al. [13] further noted that most of these issues emerged within the first year following SST and were effectively resolved. Current evidence suggested internal shield exposure may develop when positioning the buccal shield above the crestal bone during preparation and providing insufficient space between the coronal edge of the shield and the subgingival contour of the crown [13, 63]. Preventing the height of the shield from extending above the crest bone or applying a small connective tissue graft into the sulcus to assist soft tissue closure can reduce the incidence of this complications [13, 27]. Gluckman et al. [15] also recommended the creation of an S-shape prosthetic emergence profile to provide greater space for soft tissue infill. Current studies revealed no established correlation between osseointegration failure and the presence of retained root fragments. In such failure cases where the shield remained intact and uninfected, successful re-osseointegration could be achieved after implant removal and thorough site debridement followed by new implant placement. However, when the shield showed signs of infection, both the implant and root fragment required immediate removal, necessitating alternative treatment approaches [13]. It could be concluded that with appropriate preventive measures and treatment protocols, complications of SST did not substantially compromise success rates or long-term outcomes. However, future large-sample studies with prolonged follow-up were required to validate these findings and establish standardized protocols for complication treatment.

This study included both NRSI and RCT to comprehensively evaluate the comparative efficacy of SST and CIIP, ensuring broad representation of available evidence. To enhance methodological rigor, this study applied distinct quality assessment tools for NRSI and RCT and implemented systematic search strategies to minimize publication bias. Five outcomes were analyzed in this study, including horizontal and vertical labial bone resorption, implant success rate, PES and ISQ. Relevant subgroup analyses further explored the impact of critical variables on primary outcomes, including root fragment thickness, vertical positioning relative to the alveolar crest, and bone grafting in the gap between the implant and the root fragment. These subgroup analyses allowed the study to comprehensively consider the influence of various factors on outcomes following IIP or SST, providing a more systematic comparative result. By integrating heterogeneous study designs and multidimensional comparisons, this work not only clarified the mechanistic differences between SST and CIIP but also informed future research frameworks and clinical decision-making. Through this more comprehensive research design, this study could provide more precise and specific guidance for clinical practice, thereby optimizing the treatment plan for dental implants more effectively.

However, several limitations must be considered when interpreting the findings. First, although including NRSI expanded data for comparing SST and CIIP, the lower quality of NRSI compared to RCT may compromise reliability. Second, due to the specificity of the studies, blinding of clinicians was unachievable, potentially introducing performance bias. Third, the interpretation of long-term efficacy is limited by the scarcity of data from follow-up periods exceeding 12 months, compounded by a total lack of high-quality RCTs with follow-up beyond 5 years. Lastly, it should be noted that variations in treatment protocols such as bone grafting may introduce bias to the data analysis. Thus, given these limitations, the findings should be interpreted with caution, and future rigorously designed studies are still needed to validate these findings and to further investigate the long-term outcome differences between SST and CIIP.

## Conclusion

Overall, current evidence suggested that SST demonstrated advantages in reducing horizontal and vertical bone loss, improving PES, and enhancing ISQ, while showing no significant difference in implant success rates compared to CIIP. However, SST was technically demanding and required higher clinical expertise. More importantly, no consensus had been established regarding critical surgical parameters, including the ideal shield height and thickness, as well as the management of the gap between implant and shield. Although this meta-analysis provided preliminary supportive evidence for SST, the lack of standardized protocols currently limited its broad applicability. Future studies should prioritize well-designed RCTs with standardized protocols to provide reliable evidence for clinical decision-making while establishing a foundation for refining and standardizing the SST.

## Supplementary Information

Below is the link to the electronic supplementary material.

Additional file 1.

## Data Availability

The authors confirm that the data supporting the findings of this study are available within the article and its supplementary materials.

## Abbreviations

SST: Socket shield technique
CIIP: Conventional immediate implant placement
RCT: Randomized controlled trials
NRSI: Non-randomized studies of interventions
PES: Pink aesthetic score
ISQ: Implant stability quotient
MD: Mean difference
CI: Confidence interval
RR: Risk ratio
