# Supplementary material for: Clinical efficacy of socket shield technique compared to conventional immediate implant placement in the aesthetic zone: a meta-analysis
Source: Int J Implant Dent. 2025 Dec 19;11:72. doi: 10.1186/s40729-025-00657-z (PMC12717328; doi:10.1186/s40729-025-00657-z)
Supplement: Supplementary file 1 — Additional file 1. [file 40729_2025_657_MOESM1_ESM.docx]

**Supplementary Appendix**

**Page 2 Appendix Table 1** Electronic databases and search details

**Page 3 Appendix** **Fig. 1** Forest plot for the result of sensitivity analysis of horizontal buccal bone loss by excluding non-randomized studies

**Page 4 Appendix Fig. 2** Forest plot for the result of sensitivity analysis of pink aesthetic score by excluding non-randomized studies

**Page 5** **Appendix Fig. 3** Forest plot for the result of sensitivity analysis of implant success rate by excluding non-randomized studies

**Page 6** **Appendix Fig. 4** Forest plot for the result of sensitivity analysis of horizontal buccal bone loss by excluding studies with inconsistent bone grafting protocols

**Page 7 Appendix Fig. 5** Forest plot for the result of sensitivity analysis of vertical buccal bone loss by excluding studies with inconsistent bone grafting protocols

**Page 8 Appendix Fig. 6** Forest plot for the result of sensitivity analysis of implant stability quotient by excluding studies with inconsistent bone grafting protocols

**Page 9 Appendix Fig. 7** Forest plot for the result of sensitivity analysis of pink aesthetic score by excluding studies with inconsistent bone grafting protocols

**Page 10 Appendix Fig. 8** Forest plot for the result of sensitivity analysis of implant success rate by excluding studies with inconsistent bone grafting protocols

**Page 11** **Appendix Fig. 9** Funnel plot for (a) horizontal buccal bone loss, (b) pink aesthetic score, (c) implant success rate

**Appendix Table 1** The utilized Search key terms for the electronic databases

| **Databases** | **Search key terms** |
| --- | --- |
| PubMed | ("immediate dental implant loading" OR "immediate implant" OR "immediate implantation" OR "immediate implant placement" OR "immediate implant treatment" OR "post-extraction implant") AND ("socket shield technique" OR "socket shield" OR "root membrane technique" OR "root membrane" OR "partial root retention" OR "partial extraction") |
| Embase | #1: 'tooth implant':ti,ab,kw OR 'immediate dental implant loading':ti,ab,kw OR 'immediate implant':ti,ab,kw OR 'immediate implantation':ti,ab,kw OR 'immediate implant placement':ti,ab,kw OR 'post-extraction implant':ti,ab,kw OR 'immediate implant treatment':ti,ab,kw  #2: 'socket shield technique':ti,ab,kw OR 'socket shield':ti,ab,kw OR 'root membrane technique':ti,ab,kw OR 'root membrane':ti,ab,kw OR 'partial root retention':ti,ab,kw  #3: #1 AND #6 |
| Cochrane | #1 immediate dental implant loading 664  #2 immediate implant 1841  #3 immediate implantation 1197  #4 immediate implant placement 938  #5 immediate implant treatment 988  #6 post-extraction implant 157  #7 #1 or #2 or #3 or #4 or #5 or #6 2427  #8 socket shield technique 50  #9 socket shield 56  #10 root membrane technique 162  #11 root membrane 536  #12 partial root retention 62  #13 partial extraction 2909  #14 #8 or #9 or #10 or #11 or #12 or #13 3476  #15 #7 and #14 171 |
| Web of Science | (((((TS=(immediate dental implant loading)) OR TS=(immediate implant)) OR TS=(immediate implantation)) OR TS=(immediate implant placement)) OR TS=(immediate implant treatment)) OR TS=(post-extraction implant) and  (((((TS=(socket shield technique)) OR TS=(socket shield)) OR TS=(root membrane technique)) OR TS=(root membrane)) OR TS=(partial root retention)) OR TS=(partial extraction) |
| CNKI | SU=('盾构术'+'根盾术'+'根膜术'+'根片术'+‘根片屏障术’) AND SU=('即刻种植'+'即刻种植牙'+'即刻种植术') |
| VIP | 题名或关键词:(“盾构术” or “根盾术” or “根片术” or “根膜术” or “根片屏障术”) and 题名或关键词:(“即刻种植” or “即刻种植牙” or “即刻种植术”) |
| Wanfang | M=(盾构术 OR 根盾术 OR 根片术 OR 根膜术 OR 根片屏障术) AND M=(即刻种植 OR 即刻种植牙 OR 即刻种植术) |

**Appendix Fig. 1** Forest plot for the result of sensitivity analysis of horizontal buccal bone loss by excluding non-randomized studies


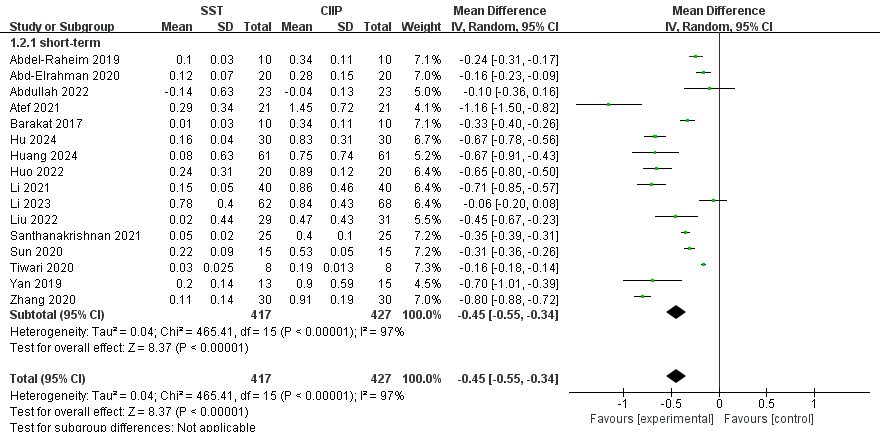


**Appendix Fig. 2** Forest plot for the result of sensitivity analysis of pink aesthetic score by excluding non-randomized studies


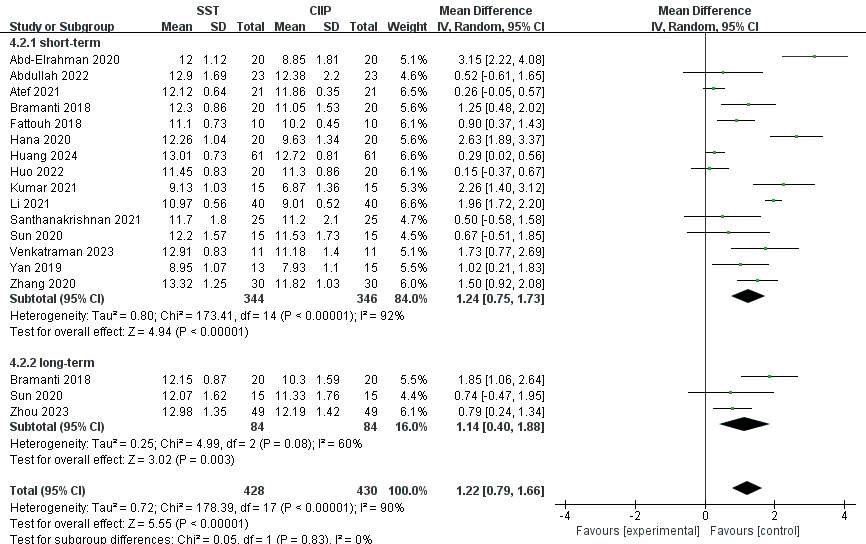


**Appendix Fig. 3** Forest plot for the result of sensitivity analysis of implant success rate by excluding non-randomized studies


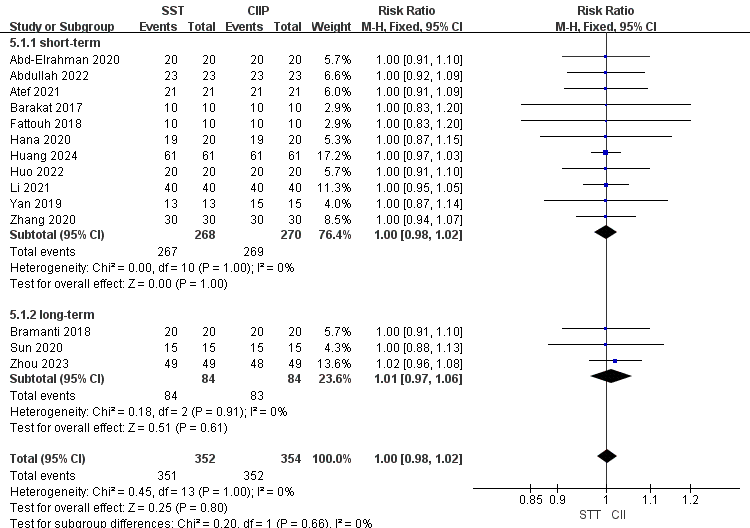


**Appendix Fig. 4** Forest plot for the result of sensitivity analysis of horizontal buccal bone loss by excluding studies with inconsistent bone grafting protocols

**
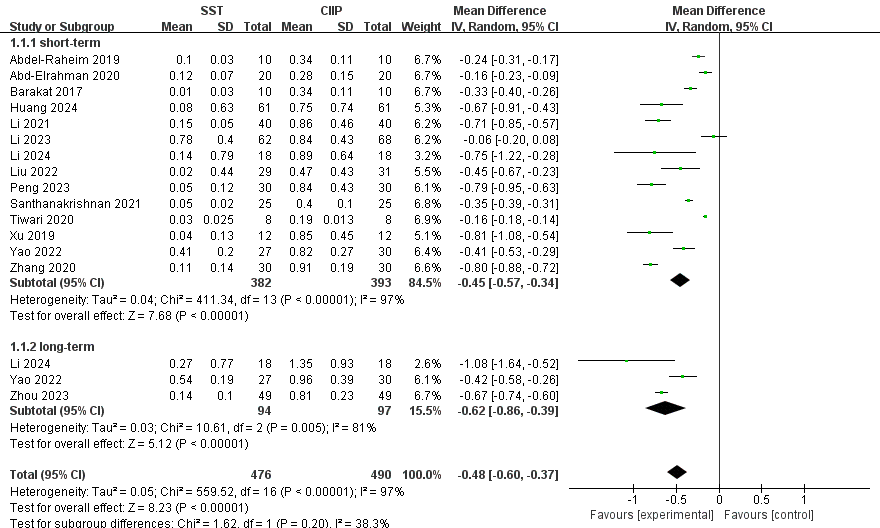
**

**Appendix Fig. 5** Forest plot for the result of sensitivity analysis of vertical buccal bone loss by excluding studies with inconsistent bone grafting protocols


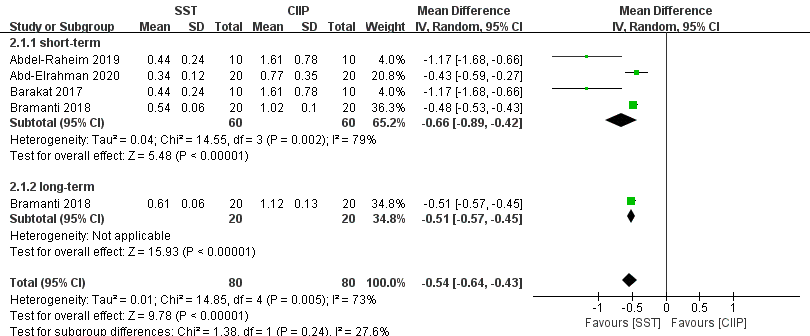


**Appendix Fig. 6** Forest plot for the result of sensitivity analysis of implant stability quotient by excluding studies with inconsistent bone grafting protocols

**
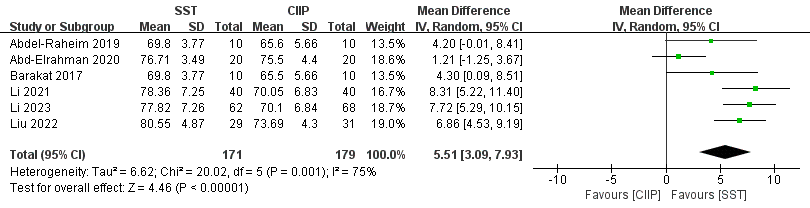
**

**Appendix Fig. 7** Forest plot for the result of sensitivity analysis of pink aesthetic score by excluding studies with inconsistent bone grafting protocols

**
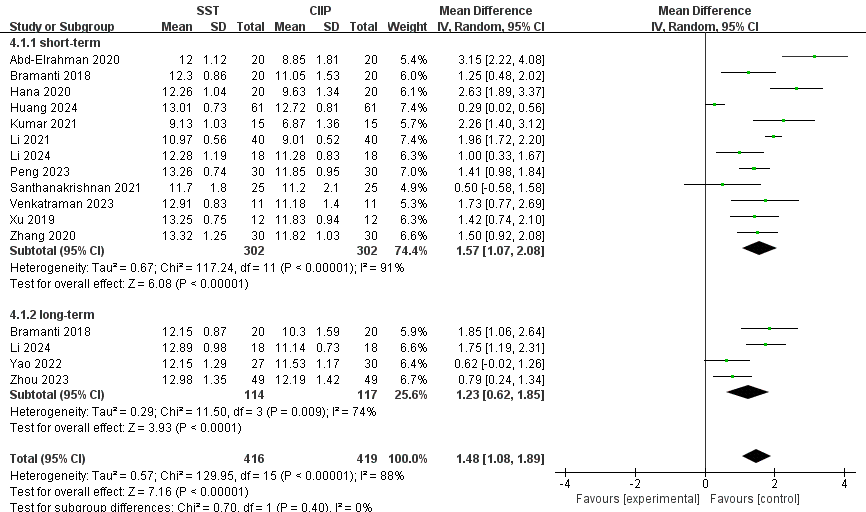
**

**Appendix Fig. 8** Forest plot for the result of sensitivity analysis of implant success rate by excluding studies with inconsistent bone grafting protocols

**
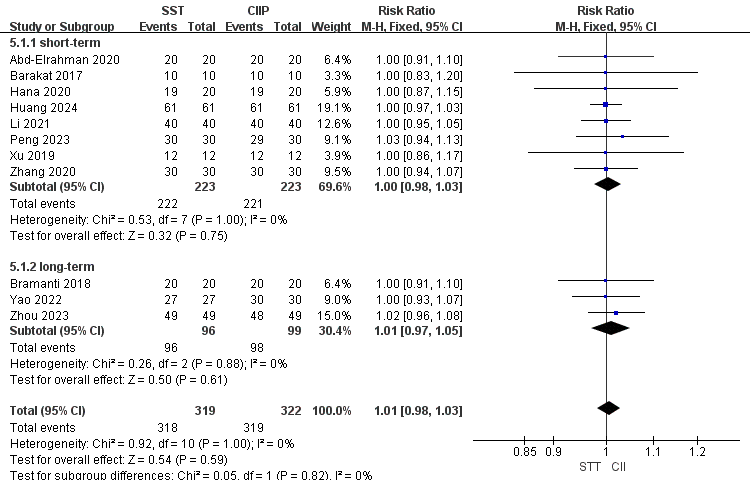
**

**Appendix Fig. 9** Funnel plot for (a) horizontal buccal bone loss, (b) pink aesthetic score, (c) implant success rate

**
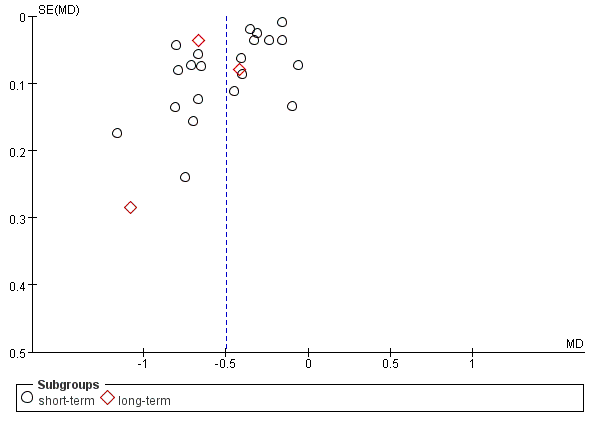

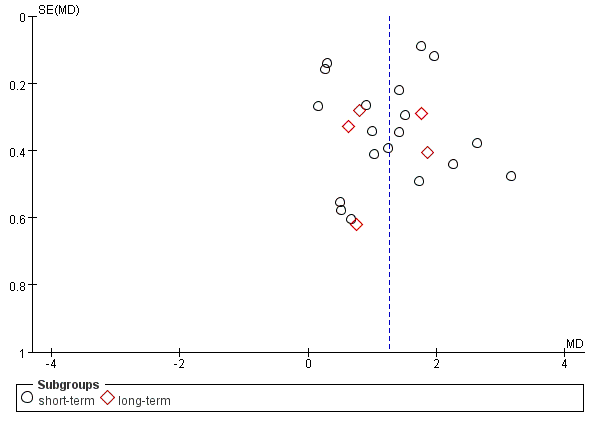

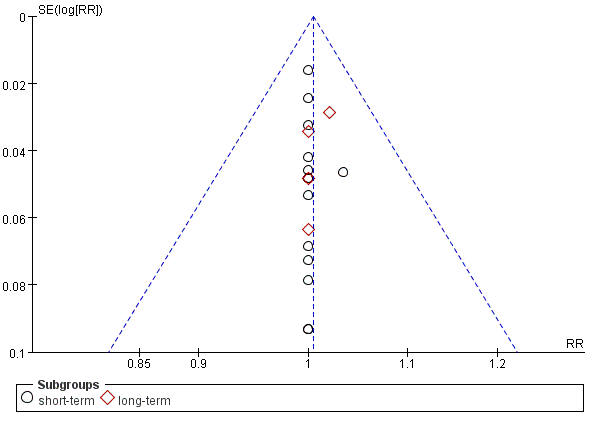
**

(a) (b) (c)
